# Data-Powered Positive Deviance during the SARS-CoV-2 Pandemic—An Ecological Pilot Study of German Districts

**DOI:** 10.3390/ijerph18189765

**Published:** 2021-09-16

**Authors:** Joshua Driesen, Ziad El-Khatib, Niklas Wulkow, Mitchell Joblin, Iskriyana Vasileva, Andreas Glücker, Valentin Kruspel, Catherine Vogel

**Affiliations:** 1 Driesen Data Analytics, 04317 Leipzig, Germany; joshua.driesen@gmx.de; 2 Department of Global Public Health, Karolinska Institutet, Solna, 17177 Stockholm, Sweden; ziad.khatib@gmail.com; 3 Department of Mathematics and Computer Science, Zuse Institute Berlin, Freie Universität Berlin, 14195 Berlin, Germany; niklas.wulkow@zib.de; 4 Siemens AG Corporate Technology, 80333 Munich, Germany; mitchell.joblin@siemens.com; 5 Iskriyana Vasileva Data Science, 10997 Berlin, Germany; vasileva_iskriyana@yahoo.com; 6 Deutsche Gesellschaft für Internationale Zusammenarbeit (GIZ) GmbH, Postfach 5180, 65726 Eschborn, Germany; andreas.gluecker@giz.de (A.G.); v.kruspel@icloud.com (V.K.)

**Keywords:** Data-Powered Positive Deviance, pandemic response, SARS-CoV-2, mixed methods

## Abstract

We introduced the mixed-methods Data-Powered Positive Deviance (DPPD) framework as a potential addition to the set of tools used to search for effective response strategies against the SARS-CoV-2 pandemic. For this purpose, we conducted a DPPD study in the context of the early stages of the German SARS-CoV-2 pandemic. We used a framework of scalable quantitative methods to identify positively deviant German districts that is novel in the scientific literature on DPPD, and subsequently employed qualitative methods to identify factors that might have contributed to their comparatively successful reduction of the forward transmission rate. Our qualitative analysis suggests that quick, proactive, decisive, and flexible/pragmatic actions, the willingness to take risks and deviate from standard procedures, good information flows both in terms of data collection and public communication, alongside the utilization of social network effects were deemed highly important by the interviewed districts. Our study design with its small qualitative sample constitutes an exploratory and illustrative effort and hence does not allow for a clear causal link to be established. Thus, the results cannot necessarily be extrapolated to other districts as is. However, the findings indicate areas for further research to assess these strategies’ effectiveness in a broader study setting. We conclude by stressing DPPD’s strengths regarding replicability, scalability, adaptability, as well as its focus on local solutions, which make it a promising framework to be applied in various contexts, e.g., in the context of the Global South.

## Key Summary Points

1. We used the mixed-methods framework DPPD (a novel approach, using quantitative and qualitative methodologies) to examine successful districts in controlling the SARS-CoV-2 transmission in Germany.
2. We identified the positively deviant districts regarding their ability to reduce the R0.
3. These districts were from two separate timeframes, the lockdown period in Spring 2020 and the 2020 summer vacation, and we conducted interviews with political representatives of some of these districts to find successful strategies for pandemic response.
4. The essential components for controlling the R0 were: fast-paced response, preparedness, situational analyses, containment, social network effects, communication and community compliance.
5. This method has potentials for low- and middle-income countries, thanks to its usage of freely available datasets.

## 1. Introduction

In December 2019, an outbreak of a novel coronavirus (SARS-CoV-2) began in the city of Wuhan, China, which was later declared a pandemic by the World Health Organization (WHO) in 2020 [1]. In the beginning of 2021, the WHO announced the approval of the first vaccine against SARS-CoV-2, under an emergency license [1]. However, there is a challenge in access to vaccination, specifically in resource-limited settings, and there is no known effective treatment for the COVID-19 disease caused by SARS-CoV-2 [1]. The virus can be transmitted fast between humans, with a basic reproductive number (R0) varying between 2 and 4 depending on the context [2,3,4]. Therefore, until an effective intervention was achieved, governments had to use so-called non-pharmaceutical interventions (NPIs) and wait for an effective vaccine or treatment in the meantime. The NPIs are meant to control the SARS-CoV-2 pandemic by reducing the R0, which requires an evidence-based, multifactorial approach: (i) reduce the human-to-human transmission, (ii) optimize testing and diagnostic services to be able to rapidly identify infected cases, and (iii) mitigate the impact of the pandemic on society by implementing control measures (e.g., using masks, practicing physical distancing, implementing policy for minimum number of persons indoors) and communicating the risk of SARS-CoV-2 to the public [5,6]. In this context, governments and public health agencies have used different approaches, due to the gap of evidence on best practices in reducing the R0 of SARS-CoV-2.

As a corollary of our main goal to introduce and showcase the novel methodology Data Powered Positive Deviance (*DPPD*) in this report, we aimed to support the search for best practices for controlling the SARS-CoV-2 transmission. To this end, we have applied DPPD on the early pandemic context in Germany. Our aim of this application was to (i) identify those German districts which managed to exceed their peers at mitigating SARS-CoV-2 forward transmissions beyond expectation, (ii) qualitatively assess the characteristics of their local pandemic response, and thus (iii) contribute to global discourse around potential best practices in this novel situation. We tested the DPPD method using multiple data sources and shared the used code and results in a public data repository [7].

### Data-Powered Positive Deviance

DPPD assumes that in every community there are individuals or groups who manage to out-perform their peers, regarding their specific local challenges [8]. It is based on the traditional Positive Deviance approach, developed by Sternin [9]. At the core of Positive Deviance is the notion that this better performance is caused by differing and uncommon practices that are more successful in and adaptive to their specific context. The Positive Deviance approach aims at identifying these practices and—if desirable and feasible—at disseminating them among the wider community [9]. As an example, Positive Deviants could be farmers, who in similar circumstances and with comparable resources achieve consistently higher yields than their peers due to differing and uncommon behaviors regarding crop cultivation. In contrast, farmers who achieve higher yields than their peers because of higher levels of precipitation would not be considered Positive Deviants, as the higher yield is not a result of specific behaviors but of climatic influences. Thus, when identifying and learning from Positive Deviants, it is crucial to consider these non-behavioral factors that affect their performance.

Albanna and Heeks [8] suggest that the increase in the availability of digital data provide an unprecedented opportunity to extend the geographical and temporal reach of the Positive Deviance approach. In other words, by using secondary data (data originally collected for other purposes), including quantitative and qualitative data, as well as “Big Data”, it might be possible to analyze whole regions and longer periods of time when looking for Positive Deviants. Secondary data can also be used to control for any non-behavioral factors that might affect performance and which therefore might lead to the false identification of Positive Deviants. For example, using data to control for the influence of precipitation on yield could help avoid falsely identifying farmers as Positive Deviants simply because their yield benefitted from higher and more frequent precipitation. Using large volumes of secondary data to search for Positive Deviants is what is referred to as the Data Powered Positive Deviance approach.

Before applying DPPD, several basic questions should be considered: Is it likely that one might find effective, differing and potentially scalable practices on the ground (scalability)? Is it likely to gain access to sufficient data to implement the method (feasibility)? Is it desirable to scale potential practices or might there be negative consequences for the wider community in doing so (desirability)?

If the conditions for the given use case are appropriate as indicated by affirmative answers to this set of questions, the method itself may be applied in two parts. In a first and quantitative part, potential Positive Deviants, the so-called *PD candidates*, are determined. For this purpose, one firstly defines the region of interest, unit of analysis and performance measure that are to be used in the analyses. Regions of interest may be individual cities or states, but also countries—as is the case in this report. The unit of analysis may be individuals, districts, regions, etc. The performance measure resembles what is termed the dependent or target variable in other quantitative procedures. It is used to identify Positive Deviants among all units of analysis. A crucial aspect of this quantitative part is to include data on and thus control for structural factors that might affect performance.

The following example can demonstrate a potential use case: one may identify Positive Deviants with higher yields among farmers. In this, one could look at individual farms or communities (unit of analysis) in an agricultural region (region of interest) and analyze their yield for a specific crop (performance measure). In addition, one might account for precipitation quantity and frequency, soil quality and temperature as factors that affect performance but are not behavioral. Controlling for these structural factors helps to identify units of analysis with similar contexts regarding their performance, the so-called homologues.

In this report, we applied DPPD to the SARS-CoV-2 pandemic in Germany (region of interest), after having answered affirmatively to the question on potential scalability, desirability and feasibility. The PD candidates were defined as those districts (units of analysis) that were able to keep the reproduction factor (R0) of SARS-CoV-2 the farthest below the levels seen in their peers, despite facing similar challenges and having access to similar resources. Heterogenous data sources, such as SARS-CoV-2 case reports, mobility and weather data were used to identify PD candidates (see Table 1 for a summary of all data sources) [10,11].

## 2. Materials and Method

The DPPD includes a two phases method that aim to learn about local and effective practices, in response to a problem the community is facing it collectively: (i) a quantitative analysis phase, and (ii) followed by a qualitative inquiry phase. Therefore, the Materials and Methods section outlines our concrete application of DPPD to the SARS-CoV-2 pandemic in Germany, explaining both the quantitative and the qualitative part of the methodology.

**Figure A1:**
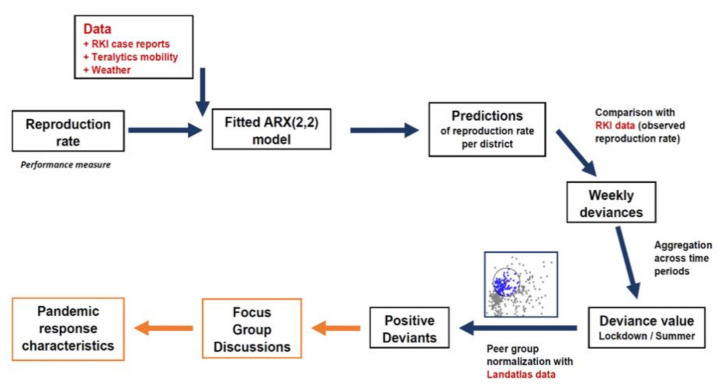
Procedural flowchart of analysis. RKI—Robert Koch Institute, ARX—auto-regressive model with exogenous input.

A graphical representation of our methodology can be found in Appendix A, Figure A1. The Materials and Methods is structured as follows: first, we described our study setting and design. This section corresponds to the step of choosing a study population in the DPPD framework. Second, we introduced our four data sources. In this section, we define both our performance measure and the confounding external factors we aimed to adjust for. Third, we described our statistical analysis, in which we generated predictions for the performance measure, aggregated the resulting weekly deviances into averages across two timeframes, and implemented the comparison between sets of homologues. And fourth, we described the qualitative procedure, in which we collected feedback from the representatives of two of the top three PD candidates for each of our two analytical timeframes.

### 2.1. Study Setting and Design

Germany has a total of 401 administrative districts, where 294 (73%) are regular counties (so-called *Landkreise*) and the remaining 107 (27%) are independent cities (so-called *Kreisfreie Städte* or *Stadtkreise*). In this article, we designated independent cities with “city of”. We chose districts as our level of analysis because it matches the spatial resolution of the publicly available SARS-CoV-2 case statistics. We treated the German capital Berlin as a single district to ensure compatibility between our different data sources.

### 2.2. Data Sources, Measurements, and Variables

We used four data sources which were obtained from publicly available sources and can be accessed through a publicly available repository, except for the mobility-related data, which are available upon request (Table 1). A set of descriptive summary statistics can be found in Table 2.

#### 2.2.1. First Data Source: Robert Koch Institute (RKI)

The RKI is a German federal government agency and research institute responsible for disease control and prevention. For our analysis, we utilised the version of the daily SARS-CoV-2 case reports published on 3 November 2020 [12], and included all cases reported between 2 March and 27 September. We aggregated these daily case reports into weekly case numbers, in order to reduce random fluctuation and to eliminate any day-of-the-week effects (such as the smaller number of tests conducted during weekends). In a next step, we estimated the weekly reproduction rate of SARS-CoV-2 (R0) in a given district by dividing the number of new cases (*NC*) in the following week (*t* + 1) by the number of new cases in a given week (*t*). To avoid divisions by zero, we added 1 to the numerator and denominator:

(1) $$R0_{t}=\frac{NC_{t+1}+1}{NC_{t}+1}$$

Finally, we transformed R0 onto a logarithmic scale to achieve a symmetrical distribution. Within the DPPD framework, this Log-R0 formed our performance measure, allowing us to compare the individual districts’ performance cross-sectionally, as well as longitudinally within one district. Choosing the R0 over other potential performance measures, such as incidences or mortality, was motivated by the following considerations: (i) the reproduction rate’s numerical interpretation does not change during the pandemic. More concretely, a value above one will always indicate an increase in case numbers, and a value below one a decrease, whereas the same incidence might be indicative of a strong local outbreak early in the pandemic but might be considered a manageable level after SARS-CoV-2 became endemic. Thus, this performance measure enabled us to compare performances more readily across different points in time; (ii) the reproduction rate’s mathematical definition offers inherent control for cross-sectional differences that might influence the incidence, such as the volume and allocation of testing. As an example, if district A always conducts 10 times as many randomly allocated tests per inhabitant as district B, this could yield as much as a tenfold incidence in district A if all cases were detected through these random tests alone. However, since this difference is present on both sides of the fraction in the reproduction factor’s formula, it simply cancels out; (iii) while mortality cases would have offered a potential performance measure which would be less affected by biases stemming from tests [13], the low total numbers of deaths during the first months of the pandemic did not allow for robust time series analysis. You can find the full data set, containing weekly infection numbers, incidences, and R0/Log-R0 values for all districts in our public repository [7].

#### 2.2.2. Second Data Source: Teralytics

Teralytics provided aggregated and anonymized mobility data derived from mobile phone call detail records from a large German network operator. The data allows an overview of the number of mobile devices performing a specific movement. For data protection reasons, Teralytics removes all information from the dataset that contains fewer than five trips on a given day. Furthermore, the data does not contain any personal information. Teralytics complies with the provisions of the GDPR and all other national and international data protection and security standards in Germany.

Within the DPPD framework, our derived mobility variables served as the first set of external confounding factors for which we wanted to control. For another study utilizing the same data set and describing its likely connection to the SARS-CoV-2 spread, see reference [14].

In our study, we used anonymized data for the period from March 9 to September 20, 2020. We aggregated these data both spatially and temporally to calculate each district’s weekly number of (a) incoming trips, (b) trips originating and terminating in the same district (i.e., internal trips and roundtrips), and (c) the sum of the incoming trips multiplied by the SARS-CoV-2 incidence in the originating districts as a measure of the incoming infection load. This aggregation removed any directional information, i.e., how many people moved between a specific pair of districts during which week, and thus provided further anonymization. In a last step, these three mobility variables were standardized by dividing them by their respective district’s population size.

#### 2.2.3. Third Data Source: German Weather Service (DWD)

Another group of factors we aimed to control for were local weather conditions. We expected the weather to have an influence on forward infection rates by changing frequency and setting (e.g., indoors vs. outdoors) of social interactions, as well as the possibly influencing aerosol persistence. To incorporate this potential influence into our predictions, we used average temperature, average humidity, average precipitation, and average hours of sunshine per day for each calendar week during the period of 9 March through 20 September 2020. The procedure and code for assigning the closest weather stations to the districts is published in our public code repository [7].

#### 2.2.4. Fourth Data Source: Landatlas

In order to identify sets of similar districts to allow for a similar comparison, we utilized both the districts’ ruralness and socio-economic status as important structural factors. These were derived from the so-called Landatlas dataset [15]. The Landatlas is part of an ongoing research project for monitoring rural areas in Germany and is being maintained by the Thünen-Institute on behalf of the German Federal Ministry for Nutrition and Agriculture (BMEL). Most of the data were collected between 2013 and 2016. Since their goal was mainly an investigation of rural areas, they only report the socio-economic status of rural districts. Thus, we adapted their methodology for creating ruralness and socio-economic status indices from their raw structural data which is available for all districts. Both indices were created by performing a principal component analysis [16] on a selection of standardized variables, taking the first component and z-standardizing it across all districts. The included variables (all continuous) in the respective indices were: settlement density, area percentage of farming and forest areas, residence percentage of one- and two-family houses, surrounding population densities weighted by distance, and distance to the next five urban centres for ruralness; and unemployment rate, mean salaries, mean income level, communal tax revenue, net population migration, residence vacancies, life expectancy at birth for men and women, and percentage of high-school drop-outs during the period of 2013–2015 for the socio-economic status.

### 2.3. Statistical Analysis

Next, we presented the details of our approach for identifying PD candidates. Our PD candidate identification was composed of six steps: (1) performance model fitting, (2) prediction of weekly expected performance, (3) calculation of weekly deviances, (4) aggregation of weekly deviances, (5) peer group normalization, and (6) ranking the districts according to their final scores.

#### 2.3.1. Performance Model

We adjusted the weekly R0 for the influence of the confounding factors mobility and weather, by fitting a regression model with these confounders as predictors, and the weekly R0 as the target variable. To account for the auto-correlated nature of the performance measure, the same district’s performance in weeks t−1 and t−2 were also used as predictors, effectively resulting in a second-order auto-regressive model with exogenous input (i.e., an ARX(2,2)) [17].

(2) $$\hat{\ln{\left(R0\right)}_{t}}=\beta_{0}+\beta_{\mathrm{AR}1}*\ln{\left(R0\right)}_{t-1}+\beta_{\mathrm{AR}2}*\ln{\left(R0\right)}_{t-2}+\beta_{X1}{*\mathrm{EXOG}}_{t}+\beta_{X2}{*\mathrm{EXOG}}_{t-1}$$

where t denotes the calendar week, ln(R0) is the natural logarithm of the weekly reproduction factor, $\beta_{0}$ is a bias factor, $\beta_{\mathrm{AR}1}$ and $\beta_{\mathrm{AR}2}$ denote auto-regressive regression weights, $\beta_{X1}$ and $\beta_{X2}$ denote exogenous regression weights and EXOG denotes the mobility and weather features of a given week.

For every district, we predicted the performance for all weeks during the period of 16 March through 20 September 2020. We used past weeks’ data as predictors, and the performance measure was calculated with regards to the following week; therefore, the model was based on data from the period between 2 March and 27 September 2020. Since data were less reliable during the beginning of the pandemic (January and February 2020), due to fewer tests and highly local outbreaks, we chose the starting date as late as possible while still containing the entire first federal lockdown. The end date was set to include all school vacations’ ending dates during the summer. The reason for the choice of the logarithm is explained in Section 2.3.3.

#### 2.3.2. Estimating Model Parameters

As a small number of extreme values can unduly influence the model parameters, our particular problem setting required a robust model fitting procedure; thanks to this procedure, we ensured that our model predictions accurately adjust for the influence of the confounders and prevent overfitting to outliers. From a statistical perspective, PD candidates can be considered a form of conditional outlier, which would be problematic for traditional squared error approaches to model parameter estimation. For these reasons, we chose Huber regression because it is known to be robust to outliers. It aims to minimize an error function that is proportionate to the squared residual for small errors and the absolute residual for large errors [18]. This way, the influence of large errors is reduced. A hyper-parameter of the model, the M-parameter, controls the switch point from squared error to absolute error. We set the M-parameter to 1.35, as this value is recommended as the optimal trade-off between robustness and efficiency [18].

#### 2.3.3. Calculation of Weekly Deviances

We applied the performance model to generate predicted weekly R0 values for all districts. These predictions corresponded to the weekly R0 we would have expected in an average district given its past performance, weather and mobility variables. Subtracting the observed R0 values from these predictions (indicated by the “hat” symbol below) yielded weekly deviance values, with positive values indicative of better performance than expected:

(3) $${\mathrm{deviance}}_{t}=\hat{\ln{\left(R0\right)}_{t}}-{\ln(R0)}_{t}$$

Using the logarithm of R0 as a performance measure, we symmetrized the range of weekly deviances around 1. As a consequence, negative and positive deviance values are equal if the quotients between true and predicted R0s are equal, instead of the absolute deviations (since log(a) − log(b) = log(a/b)). For example, let the predicted R0 of a district be 1.5 and the true R0 equal to 2 in one week. In the following week, the district manages an R0 of 0.75 while the predicted R0 was 1. Then, we find that the average deviance for these two weeks is equal to 0 since the district followed an underperformance by 25% with an overperformance by 25%. Note that, had the true R0s been equal to the predicted ones in both weeks, the deviance over both weeks would have been 0 as well, although the total number of case numbers would have been lower. However, we chose the logarithm since we strived to evaluate a district’s average success or failure to react to the current situation instead of the overall performance across a timeframe.

#### 2.3.4. Aggregation of Weekly Deviances

The resulting deviances followed a roughly symmetrical distribution across all district weeks. We achieved a manageable number of between-district comparisons by flattening the temporal dimension of our timeseries data. To this end, the weekly deviances were aggregated by averaging them across two timeframes for further analysis, resulting in one value for so-called *lockdown deviance* and one value for so-called *summer vacation deviance* for each district:

(4) $${\mathrm{deviance}}_{\mathrm{aggregated}}=\frac{1}{T}\overset{T}{\underset{t=1}{\sum }}{\mathrm{deviance}}_{t}$$

Here, each timeframe was indicated by a range of weeks from 1 to T, with T corresponding to the timeframe’s length in weeks. This reduction of complexity necessarily involved a certain decrease of informational content, as the aggregation no longer differentiated between, e.g., two districts where one does not deviate from its respective prediction at all, whereas the other always deviates by the same constant amount in alternating directions. However, this reduction was necessary so we could obtain a manageable number of district rankings, and thus PD candidates.

The choice of these particular timeframes was motivated mostly by the public perception of these two timeframes being unique in regard to the set of potential policies: The first lockdown introduced unprecedented infringements into personal liberties, while during the summer vacation, people experienced a “new normal”, and only minimal restrictions remained in place.

For the lockdown deviance, we used one fixed time interval, starting from March 16 (calendar week 12), in which the German Chancellor Dr Angela Merkel announced the federal lockdown in a televised address. Defining an endpoint for the first lockdown in Germany proved more complex, as different restrictions were lifted at different times. We chose May 24 (calendar week 21), as this was the week when all restaurants and hotels in Germany could reopen [19].

For the summer vacation period, the dates for the schools’ summer vacations differ between German provinces. Since we assumed vacations to have a strong effect on the dynamic of the pandemic, comparing the same timeframe across districts with different vacation periods might have led to a biased comparison. Therefore, we aggregated different timeframes for different districts depending on their province: a given district’s summer vacation deviance was set as the average of the seven-week interval beginning with the calendar week of its province’s first day of school summer vacation. Usually, summer vacations in Germany last for only six weeks, but we added one more week to ensure that all travel returnees’ potential infections would have taken effect.

#### 2.3.5. Peer Group Normalization

**Figure A2:**
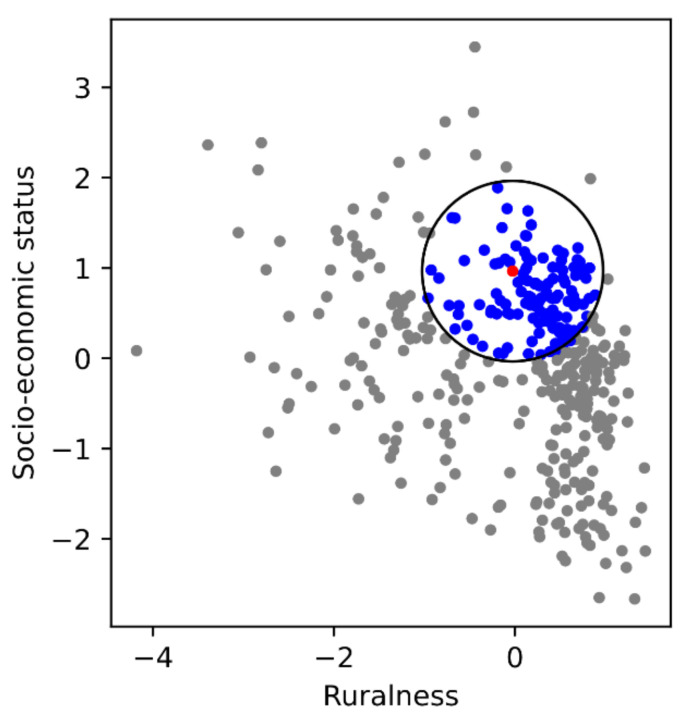
Peer group identification for one example district (city of Koblenz). Red dot represents district for which peers are to be identified, blue dots represent peer districts, grey dots represent districts not considered during peer group normalization for this particular district.

After aggregation, there were now two deviance values per district. However, comparing these directly would not take into account that different kinds of districts might face different challenges due to structural factors such as population density or social factors such as unemployment. To avoid this, one key element of the DPPD method is to define sets of homologues for which ‘fair’ comparisons can be made. In our study, we used the indices of ruralness and socio-economic status described earlier to identify these homologues, as these factors likely influenced which policy targets were available (e.g., mandatory masks in subways are only possible in urban centres) and feasible (e.g., higher socioeconomic status means more jobs that allow working from home [20]). Moreover, structural aspects such as the settlement density might directly influence the baseline reproduction rate. Since these district properties are static during the period of analysis and are thus unable to explain within-district variation, we did not include them as predictors in our timeseries model but used them to find homologous districts. Instead of creating a fixed set of homologue clusters, each district was assigned its own set of peers. Using the indices for ruralness and socio-economic status as x- and y-coordinates, two districts were considered peers if they were within a radius of 1 of each other. We chose this radius because it corresponds to one standard deviation of the indices. Figure A2 (in Appendix A) illustrates the peer group identification for one example district.

Each district’s aggregated deviance values were then normalized relative to its respective peer groups according to the formula:

(5) $${\mathrm{deviance}}_{\mathrm{normalized}}=\frac{{\mathrm{deviance}}_{\mathrm{aggregated}}-\mu_{\mathrm{peer}\;\mathrm{group}}}{\sigma_{\mathrm{peer}\;\mathrm{group}}}$$

where µ and σ indicate the peer group’s mean and standard deviation, respectively, with the focal district included in their calculation.

#### 2.3.6. Ranking of Districts

In a final step, we used the aggregated and peer-group normalized deviances to rank the districts according to how much they exceeded both our expectations given their prior performance, weather, and mobility, and how much they exceeded their peers in that regard. According to the DPPD framework, the likelihood of finding effective strategies should be highest when these final deviances are highest. Therefore, we chose the three best performing districts for each of the two aggregation timeframes for an in-depth strategy search using focus group discussions (FGDs).

### 2.4. Focus Group Discussions with PD Candidates

After identifying the highest-ranking districts as PD candidates for each timeframe, we conducted semi-structured FGDs with a selection of potential PD candidate districts. Overall, six districts were invited, of which four districts participated: the city of Koblenz and Lörrach for the first lockdown and Gütersloh and Vechta for the summer vacation period (Table 3). Two districts’ representatives chose not to take part due to high workload. The discussions were structured using an interview guide, which was tested in a pilot interview with a non-PD district and subsequently refined and finalized.

We performed four virtual FGDs—one per participating district—in the period between 1 February to 25 March 2021. The focus groups were made up of two to three participants per district/interview from various departments in the local administrations that all held key positions in shaping their district’s respective pandemic response. Furthermore, in addition to the interviewer, one or two more research team members joined the videoconferences to take notes and capture impressions about the meeting.

The aim of the FGDs was to identify factors, guiding principles, and actions that likely contributed to the respective district’s positively deviant performance during the respective timeframes. Therefore, we were particularly interested in what these districts considered to be the success factors in their fight against the pandemic, with a special focus on differences between their own and potential ‘non-PD’ pandemic responses.

In accordance with the written consent granted by all interviewees, the discussions (60–90 min per meeting) were recorded and subsequently transcribed. Next, we coded and categorized the resulting textual data. The FGDs were conducted in German, as was the transcription and coding. The final results were translated into English. We performed the qualitative analysis using the MS Office Suite.

Handling, storage, and deletion of personal data in the form of the recordings have been conducted in line with all relevant data protection regulations. Participants were informed of these aspects and of their opportunity to refuse their consent or retract it at any later point in time. GIZ’s legal and data protection units were consulted from early on to ensure compliance with the highest ethical and legal regulations.

## 3. Results

### 3.1. Regression Model

Our ARX(2,2) regression model with minimized Huber loss was able to account for 14.76% of the weekly log-reproduction factors’ variance. To ensure a sufficient model fit, we investigated the Pearson correlations between each predictor and the predictions’ residuals. All correlation coefficients were between −0.01 and 0.02, indicating that our model successfully removed all (linear) dependencies of our confounder variables and the resulting deviance. For the resulting regression weights and information about their interpretation, see Appendix B.

### 3.2. PD Candidates

**Figure 1:**
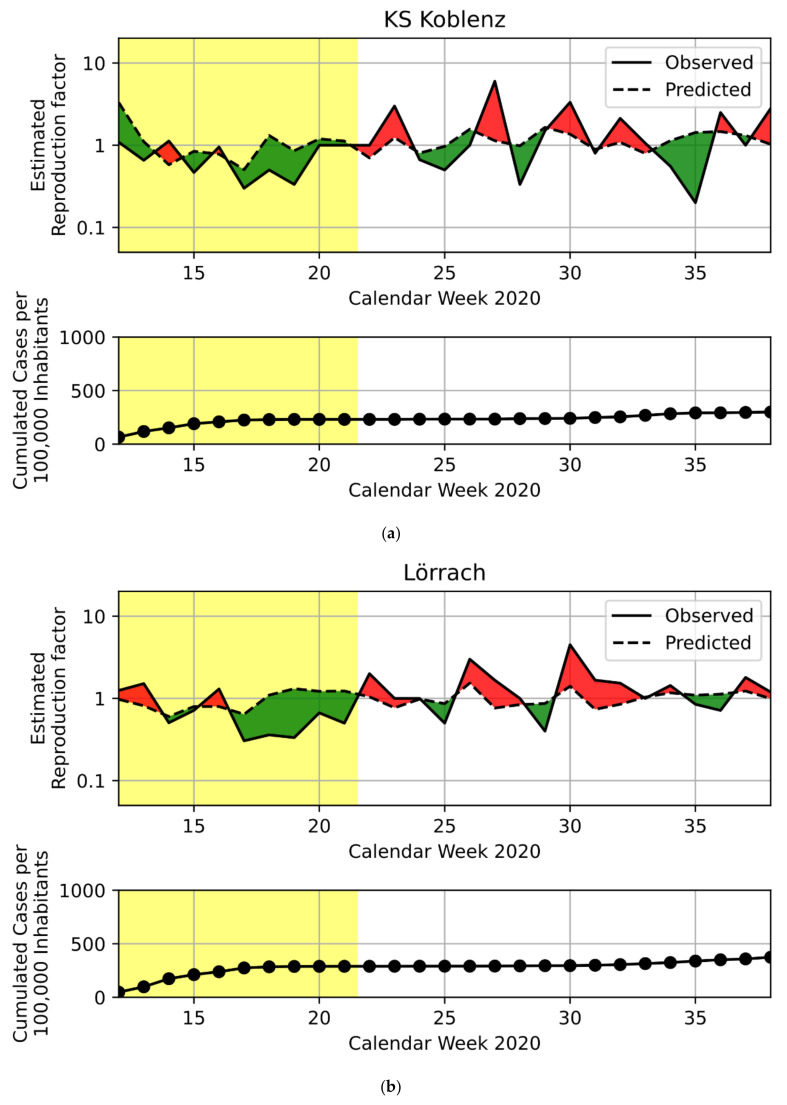

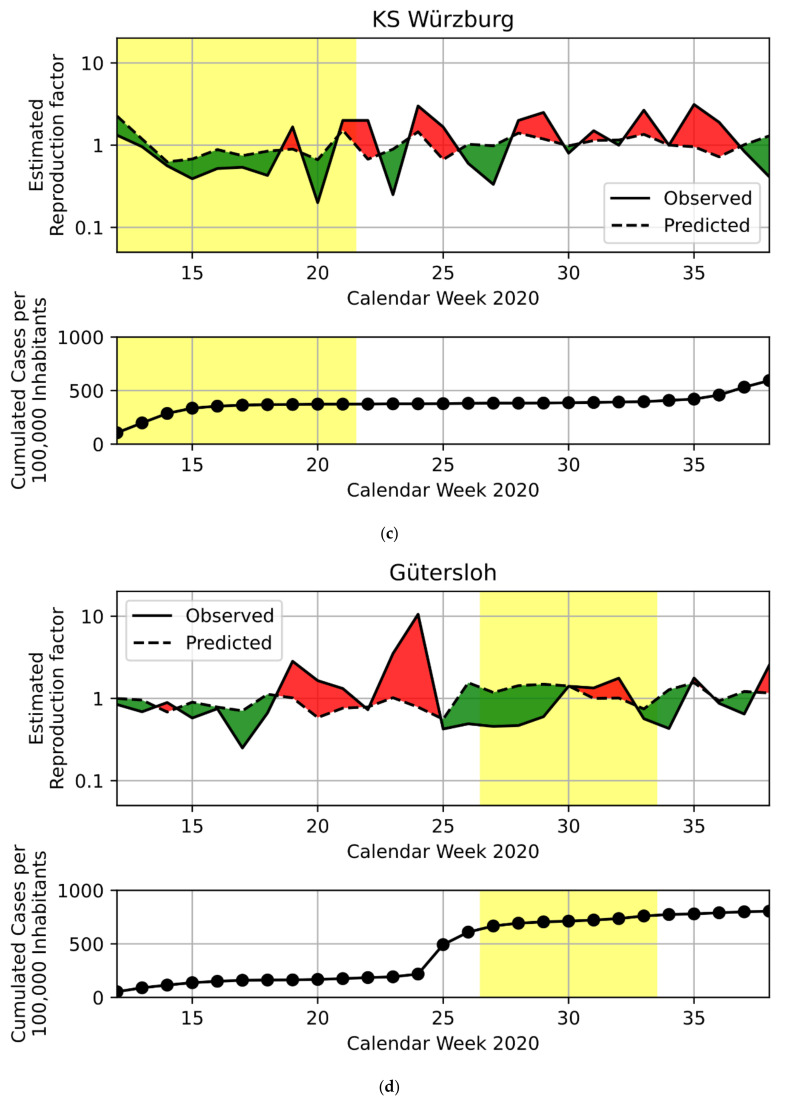

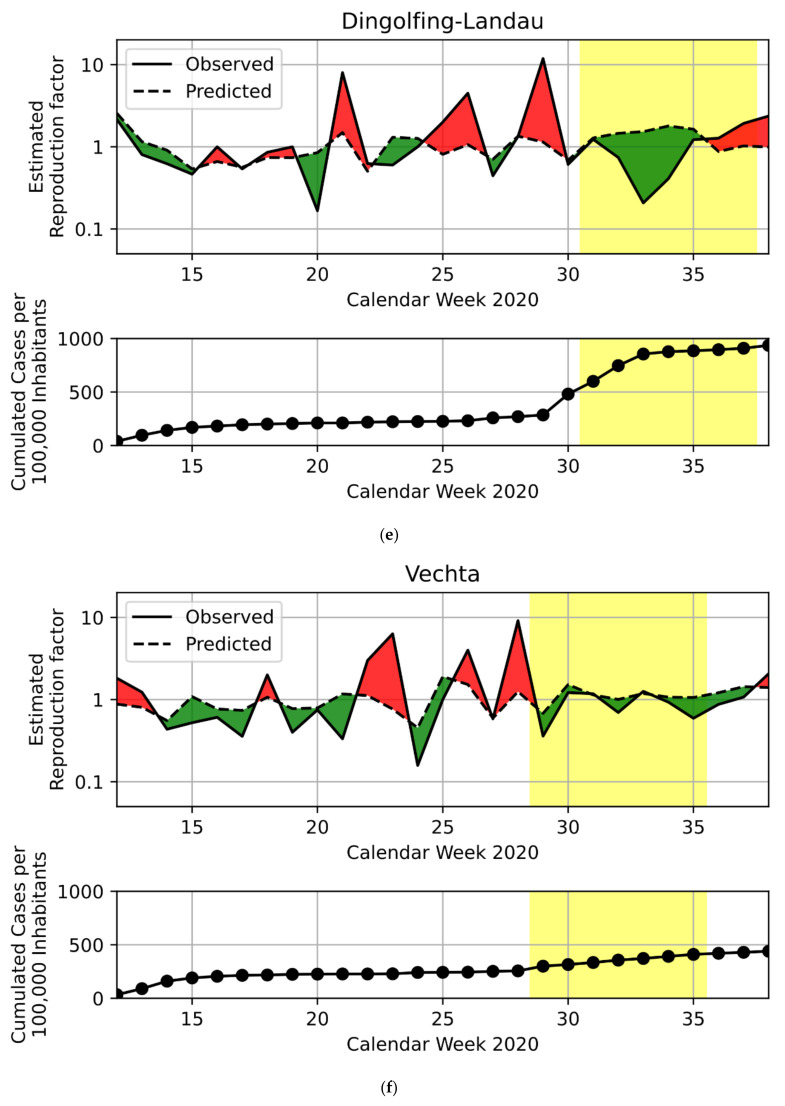

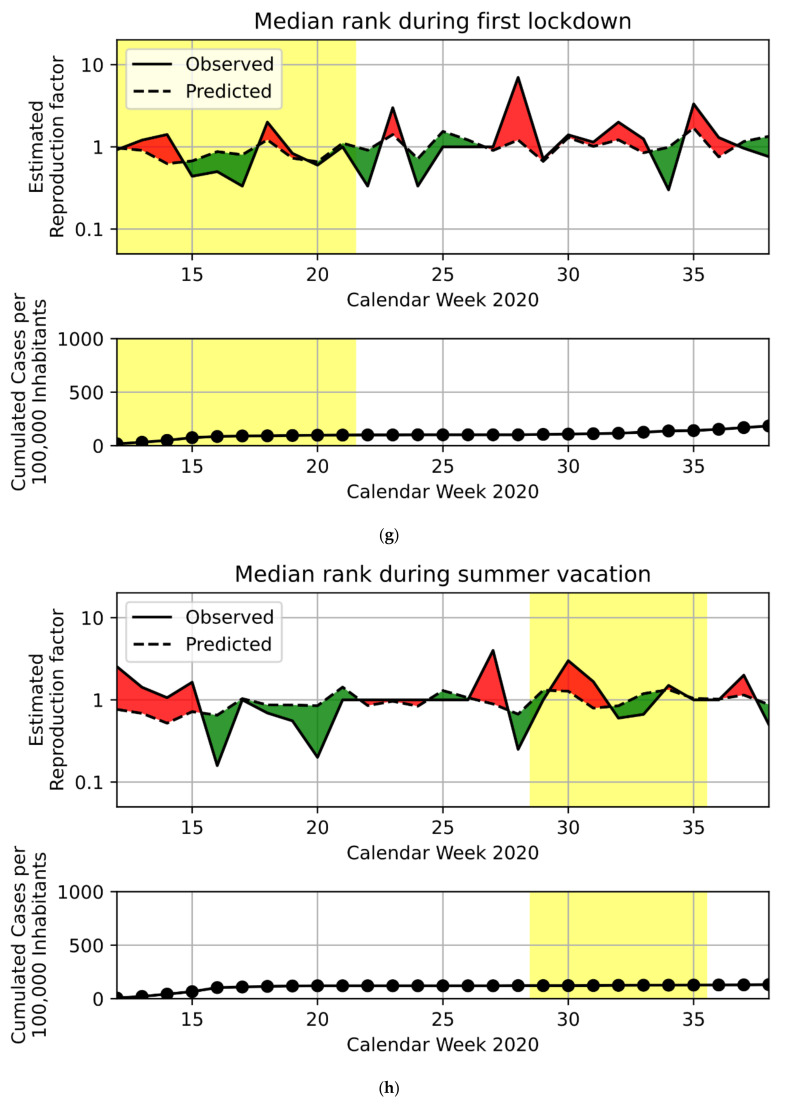

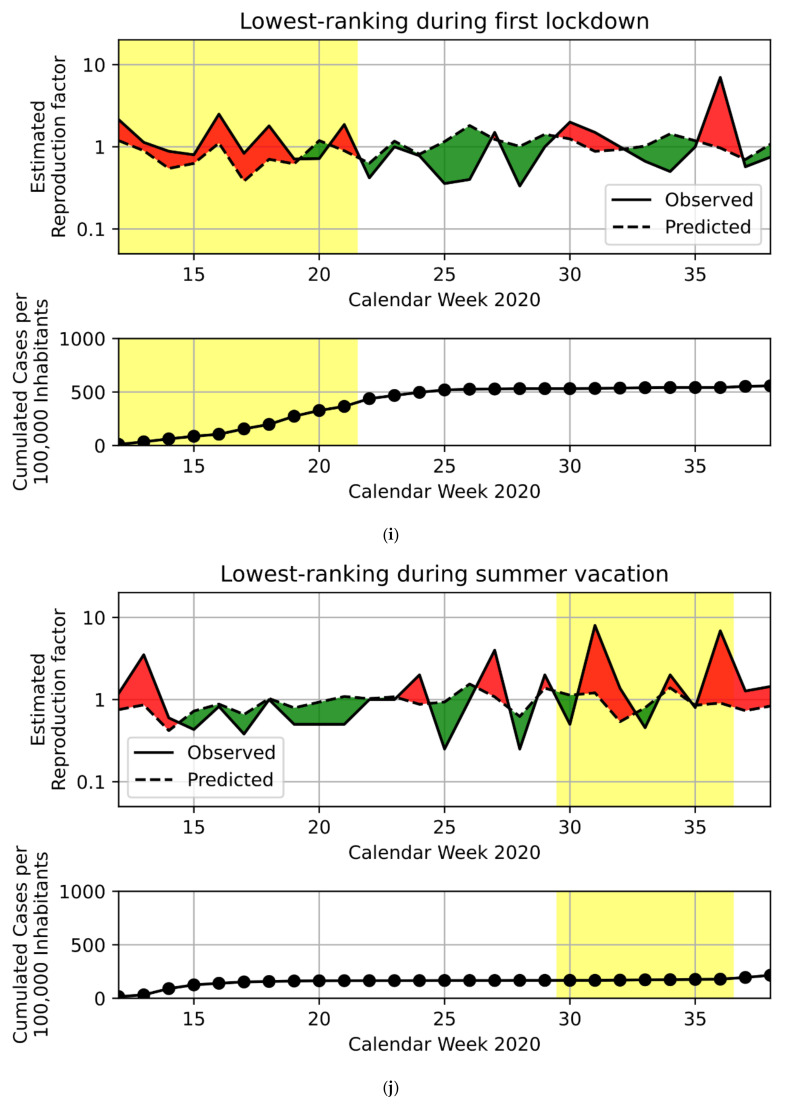
Reproduction rate and cumulated incidence of selected German districts. In descending ranking order, (**a**–**c**) show the identified PD candidate districts for the first lockdown, and (**d**–**f**) show those for the summer vacation period. For comparison, we also show each timeframe’s median (**g**,**h**) and lowest-ranking (**i**,**j**) districts. Yellow shading indicates the timeframe for which the district was identified as either PD candidate, or median/lowest-ranking comparison district. Upper sub-plots show both the predicted (dashed line) and observed (solid line) weekly reproduction factor on a log-scale. Green areas indicate positive deviance values where the observed values were lower than predicted. Red areas indicate negative deviance where the observed values exceeded the predictions. Since our model utilized log-R0′s, the red and green areas correspond to actual deviance values despite the log-scale axis. Lower sub-plots show cumulated case numbers per 100,000 inhabitants, included to visualize the relationship of forward transmission and case incidence. KS = ‘Kreisfreie Stadt’, i.e., the independent city districts explained in Section 2.1.

According to our ranking, the three districts with the highest positive deviance during the first lockdown were, in descending order: the city of Koblenz, Lörrach and the city of Würzburg. During their respective summer vacation periods, the three highest-ranking districts were, again in descending order: Gütersloh, Dingolfing-Landau and Vechta. See Table 3 for a description of these six PD candidates. Figure 1 shows these districts’ predicted and observed reproduction factors, as well as their cumulated incidences. For both the first lockdown and the summer vacation period, we also show the same plots for the median and lowest-ranking districts, to facilitate the interpretation of the PD candidates’ performance. The representatives of four districts accepted to participate, while the representatives of two districts, Dingolfing-Landau and the city of Würzburg, declined. Thus, the following qualitative results stem from the FGDs with representatives of the city of Koblenz, Lörrach, Gütersloh and Vechta.

### 3.3. Focus Group Discussion

When harmonizing and analyzing the qualitative data from four FGDs, a total of seven themes emerged that all contain relevant information regarding factors and strategies that interviewees felt contributed to their positively deviant pandemic response. All seven themes, as well as accompanying codes, are displayed in Table 4. Citations were included in the text below. Before translation, duplicates and filler words were removed for legibility purposes. The content and meaning were not altered in any way. Empty square brackets with three dots indicate text omissions other than duplicates and/or filler words. Text in square brackets was added to provide relevant contextual information. Subsequently, we described the individual themes in more detail.

#### 3.3.1. Theme 1: Characteristics of Local Administrations’ Fast-Paced SARS-CoV-2 Emergency Response

##### Action Bias

FGD participants described a prioritization of proactive actions, which they contrasted with administrative processes that in their views may at times be characterized by longer ‘waiting times’. Being in a proactive mode, instead of waiting for guidance, was described as the preferred modus operandi. Furthermore, discussions about administrative responsibilities moved into the background.

*“But that is precisely the point, acting flexibly and pragmatically, looking ahead and staying ahead of the situation, as you say in crisis response circles. I believe this to be a very important thing. Instead of doing the usual bureaucratic waiting and seeing what happens and what we guidelines we get.”*

*“I believe it is important to always think proactively, and not first wait until you get some guidelines from [federal or provincial institutions], but instead react to the local situation on a daily basis.”*

*“The [citizen] doesn’t ask, whose responsibility is this, he asks how he can be helped.”*

*“[Some task] was basically not the health department’s job, but we just did it, because we saw that it needed to be done.”*

##### Agility in Public Administration

The interviewees stressed the local governments’ increased agility in response to the pandemic. They reported on their perception of their respective administrations as being characterized by high levels of effectiveness, flexibility and adaptability. This was also described on the level of individuals in the administrations, whose high levels of enduring motivation were reported to be an essential success factor.

*“If you had asked me two years ago whether I can imagine a health department as an agile organisation, I would have said ‘What? No way’. And now, after this year, yes, I have never seen a more agile organisation than a health department.”*

*“Then naturally, you need people who are highly committed, who stay with it and no one says ‘It’s the weekend now’.”*

##### Fill Regulatory Gaps/Courage to Deviate

The participants emphasized the will and courage to take difficult decisions when there were gaps in official guidance and/or where opinions by different stakeholders varied.

*“Wherever possible we wanted to act ourselves and shape the response, and simply act. Of course, we got quite a lot of, let’s say, scepticism or first astonishment. But in the end, they [the decision makers higher up in the hierarchy], noticed that we always fared well with our strategy, and actually were always acting proactively, which turned essentially always turned out well.”*

*“We were always [operating] on a daily basis, as soon as we noticed that the Robert Koch Institute changed its guidelines, we immediately implemented them, and everything for which there was no guideline, we just amended it reasonably.”*

*“There was no [pandemic response] blueprint in Germany. We had to develop it ourselves in the shortest possible time. We developed it using our experience with normal containment plus the special [characteristics of] the situation.”*

##### Cross-Organizational Collaboration

In all FGDs, participants emphasized the importance of cross-organizational collaboration between different groups of local stakeholders. Participants from all interviews stressed that joint efforts were crucial in tackling the pandemic. One important aspect that emerged from the interviews was short communication lines, enabled by collaborative structures such as task forces and crisis management groups.

*“It is absolutely imperative that ‘silos’ that normally operate entirely side by side, in this kind of situation work hand in hand, and coordinate with each other very closely.”*

*“[…] and having, as is the purpose of a crisis management group, relatively short communication lines. Thus, the reported situation as we appraised it was known to everyone.”*

#### 3.3.2. Theme 2: Preparedness

##### Risk Consciousness and Early Initiation of Measures

Across all FGDs, participants talked about preparedness in relation to the timeframe of the first federal lockdown. Interview partners described high levels of risk consciousness in their respective districts, which they explained with their awareness of the situation in early hotspots, available expert knowledge and the structural characteristics of their respective districts. Additionally, the participants described that their high level of risk consciousness led them to plan and implement structures they deemed necessary to tackle a potential outbreak as soon as it would eventually emerge.

*“We saw what happened in China. We saw what happened in Italy, and it was absolutely clear: This will come to us—and when it happened, we acted very quickly and resolutely implemented measures which we deemed reasonable under medical aspects for containing a pandemic.”*

*“We have to generate a road map and we have to be ready to go, when the first case happens in our district, we have to have created administrative structures.”*

##### Emergency Infrastructure and Procurement

Besides relevant organizational structures, interviewees stressed the importance of setting up necessary emergency infrastructures and procuring extra goods to be prepared for potentially severe infection dynamics. Interviewees described that these infrastructures were not needed in all instances; however, they still considered them an important asset to have if worst-case scenarios had occurred. Additionally, media coverage of these preparations served to increase the risk awareness among the general population.

*“In total, we built three facilities […] with almost 340 beds. Also, nurses would have been available, and we would have ensured 24-h operation. We were prepared.”*

#### 3.3.3. Theme 3. Situational Analysis and Reporting

##### Data Flow

Participants reported that effective and efficient data flows were an important factor in their districts. Collecting relevant data quickly and ensuring the flow of data between relevant stakeholders, as well as centrally aggregating and processing it further emerged as important aspects from the interviews.

*“We from the emergency services contacted [local policy makers] and said: We now need a team for continuous situation monitoring, i.e., we used the emergency services for this; all reports coming in from the city need to go into one central department, and the politically responsible need to examine them and generate a [response].”*

##### Analyses and Projections

The participants reported that processing the available data to make it actionable, mainly by producing holistic yet granular analyses of the status quo, and projections that can show potential trends played an important part.

*“We have a daily data analysis. In this data analysis, in a very detailed form, we started very early, but extended it further in the context of the outbreak, in order to assess very precisely whether we have infections into sensitive areas like schools, day cares, and care facilities.”*

##### Information Technology (IT) System in Health Departments

Most participants mentioned the importance of a dedicated IT system that helped health departments with data handling and reporting, but also with contact tracing and other containment efforts. Various interviewees stressed the importance of having invested time and resources in ‘upgrading to’ such a dedicated system early in the pandemic.

*“A special software was introduced, which is still in use today, to handle and track the cases. However, we were also able to assign big IT-based teams with special software, where we could monitor everything, and also send out our directives.”*

#### 3.3.4. Theme 4. Containment

##### Strive to Remain in Control

Across all four FGDs, participants reported the high importance of remaining in control and staying ahead of the situation whenever possible or returning to this state as quickly as possible.

*“Therefore, we acted quickly and created the fever walk-in clinic […] to stay ahead of the situation and keep the infections as low as possible, identify early on who is infected, identify contacts and isolate accordingly.”*

*“We always had the aspiration to keep the situation under control, and I believe we also had the situation under control during times when we had a higher infection occurrence, and we were working every day of the week.”*

##### Shut Down and Isolate Early and Comprehensively

All study participants shared that they implemented early measures to contain the spread of SARS-CoV-2, which shut down public life and other affected sectors, as well as isolating identified cases and quarantining close contacts quickly and comprehensively. While the individual actions taken (e.g., which institution to close when) differed between districts and timeframes, this mode of implementing these particular containment steps early and decidedly was present across all FGDs.

*“We started very early on to shut down public life through [a far-reaching policy instrument].”*

*“We were always very courageous in our decision. We did not isolate individual [school] classes, but also said: the institution gets shut down. No one had a whole lot of experience, but we acted aggressively—always stay ahead of the situation.”*

##### Large Scale Testing

All participants revealed that the represented districts employed large-scale testing to accompany other containment efforts such as shutdowns/lockdowns. Interviewees also shared their perception that more tests were conducted in their respective districts than in surrounding areas. Several districts reported that they implemented specific testing regimes several weeks prior to the initiation of national testing guidelines.

*“This was always our strategy: testing, testing—targeted of course, but also a lot.”*

#### 3.3.5. Theme 5. Communication

##### Reliable and Trustworthy Public Communication

The focus group participants stressed the importance of reliable and trustworthy public communication and investing the necessary resources to achieve that goal. Some interviewees felt that this was a dimension that had not received enough attention in other districts.

*“Every crisis needs a face. […] Daily video messages, two to three minutes: What is happening here? Why are we doing this? Why are we ahead of the situation? What are the rules?”*

*“But many neglected the media situation, that needed to be handled comprehensively. Early on this took almost as much time as the medical situation.”*

##### Consistent Health Messages Dissemination across All Channels

The interviewees shared that their respective districts used various communication channels to give frequent updates and communicate and explain decisions (e.g., on potential restrictions). They shared that the public reacted very quickly to inconsistencies and differences in wording between the different information sources and channels. Therefore, ensuring congruent messaging across all channels was deemed important.

*“[It is important] to speak with one voice. We often noticed that, whenever there were small differences in wording between [different communication channels], people were very preoccupied with this, so we learned to coordinate our publications very well.”*

#### 3.3.6. Theme 6. Utilization of Social Capital

##### Personal Networks of Decision Makers

The participants emphasized that many decisions and actions were accelerated by good personal networks of decision makers.

*“And again, one thing was helpful, […] having a [senior health dept. official] who knows the district and knows everyone here. Compared to a someone where this is not so, this is an important factor, I think.”*

##### Role of Social Cohesion

The role of social cohesion and overall good connections between the districts’ inhabitants was mentioned as an asset when confronting the pandemic.

*“We have about 23,000 members of carnival associations. We then started an appeal, since one knows one another, one is in exchange with one another. Many associations sewed face masks, which in part came to us, and in part were distributed within those associations.”*

*“On one hand, we’re a district with international players […]. On the other hand, we have connections like in a small village—one does know one another. And this is totally helpful for contact tracing, even though they are actually global players. […] One knows one another and can utilize personal, informal channels.”*

#### 3.3.7. Theme 7. Community Compliance

##### Early Preparedness Measures Reinforce Risk Consciousness among the Population

The data underlines that community compliance with existing pandemic regulations, as well as the factors positively influencing this compliance, may have been a factor that contributed to the districts’ positively deviant outcomes. Interviewees from FGDs covering the first lockdown emphasized that early preparedness measures reinforced the popular recognition of the pandemic as real and dangerous, which in turn may have led to higher compliance with existing measures.

*“We noticed that we didn‘t need a lot of [built emergency infrastructure], like overflow hospitals. But it was covered in the media, which of course created some sensibility in the population.”*

##### Proximity of a Major Outbreak Increases Compliance

Interviewees from the summer vacation timeframe stressed that the local proximity to major outbreaks that took place in their respective districts around the beginning of the summer vacations may also have increased compliance among the population. This effect was also evident in the mobility data, wherein a marked reduction in individual trips occurred in the weeks after the respective outbreak was reported on in the national news. While our statistical model already accounts for this mobility-specific aspect of compliance, it seems likely that it was accompanied by other voluntary behaviour changes not captured in this data.

*“I think there was generally high sensibility in the population here. That must be said very clearly. We think that the [distancing, hygiene and mask] rules had a higher significance due to [the local, publicised outbreak], compared to regions where it was a lot easy-going after the first wave.”*

## 4. Discussion

We implemented a novel mixed-methods approach DPPD in the context of the SARS-CoV-2 pandemic in Germany. We identified a total of six positively deviant German districts across two timeframes, after adjusting for prior infection spread, mobility and weather influences, and ensuring fair comparisons between structurally similar districts. On average, these districts managed to keep the R0 of SARS-CoV-2 furthest below the expected level during these two selected timeframes, thus successfully limiting the (further) transmission of infections, effectively flattening the curve in their respective districts.

Making use of Big Data to identify the PD candidates, combined with qualitative interviews with local policy makers from four of our six PD candidates was a useful approach to understand the factors and practices that may have contributed to their positively deviant performance during the two investigated timeframes [21]. The main advantage of this mixed-methods approach was to leverage the scalability of quantitative methods to allocate the more labor-intensive qualitative methods to the most promising districts, thereby achieving higher efficiency for the identification of successful strategies.

### 4.1. Pandemic Response Characteristics of the PD Candidates

The qualitative results presented above show a high degree of parallels in factors that were deemed important for a successful pandemic response during the given timeframes across the four interviewed districts. The identified themes were present in all interviews, with most codes/subcategories also being present in the majority of district responses. The themes mainly describe guiding principles that seem to have influenced certain actions rather than describing a concrete set of measures themselves. The shared nature of these guiding principles across the interviews indicates that there seems to have been a set of shared ‘key tenets’ the districts deemed crucial for their successful pandemic response.

However, before assessing and describing these ‘key tenets’ in more detail, subsequently, one also has to stress that there were noteworthy differences in the interviewed districts’ pandemic responses. Most of these differences can be found on the level of concrete actions. These differences may be traced back to different stages of the pandemic for which the respective districts appeared positively deviant, as well as context-specific events taking place at this time: in the first timeframe, the focus was on being prepared for the emergence of the first cases in the respective districts. In contrast, during the summer holiday season, the focus was on detecting and containing local outbreaks as they occur. Interestingly, both summer vacation PD candidates for which we conducted FGDs experienced a major outbreak in a meat-processing plant just prior to the relevant timeframe. For a detailed discussion of the outbreak in Gütersloh, see references [22,23]. The localized nature of these outbreaks, with meat-processing workers forming relatively close-knit ‘quasi-closed’ communities, may have enabled the local administration to effectively contain these outbreaks and keep them from affecting the general population.

However, as noted before, while clear differences exist on the level of concrete steps taken by the districts, the guiding principles that emerged from the interviews and that are described in the seven themes presented above are mostly consistent throughout all interviews. Interestingly, the characteristics that describe theme 1 are also explicitly or implicitly present in many of the other themes. The tendency to move forward preferring quick and decided actions over longer waiting periods, despite the many unknown factors of the novel situation that can, for instance, be found in preparedness efforts (theme 2), efforts to set up an infrastructure for situational analysis and reporting (theme 3) and containment efforts (theme 4). The proactive actions taken by the districts were often also accompanied by a general high degree of flexibility both in terms of adapting to quickly changing circumstances and in combination with the aforementioned proactivity when opting to potentially deviate from standard procedures in order to adapt their response to the local context. This often took the form of implementing quicker and/or stricter preparation (theme 2) and containment measures (theme 4) than what had been instructed from the provincial or federal level. Furthermore, the importance of cooperation between different stakeholders within and outside of the public administration was also present in most themes and was constantly stressed by all interviewees. The presence of the four principles described in theme 1 across the majority of themes across (almost) all interviews suggest that they functioned as a kind of overarching guiding principles that seem to have played an important part in shaping our PD candidates’ respective pandemic response.

Next to a general fast-paced, proactive, and flexible response, allocating resources to improved information gathering, as well as coherent communication with the public, were deemed highly important as well. This matches prior research showing clear, consistent, and coherent public communication to be beneficial for community uptake of pandemic response measures [24,25,26].

Furthermore, our qualitative results stress the importance of social network effects, both in the sense of social cohesion in the public, and in the sense of personal networks facilitating a speedy response [27]. These findings overlap with previous studies, wherein proper risk communication and social capital were shown to contribute to the improvement of risk prevention, in a calm way [28], by communities during major global health events including SARS-CoV-2 [28,29], severe acute respiratory syndrome (SARS), Middle East respiratory syndrome (MERS), influenza A(H1N1), Ebola [30,31,32,33], and other stressful situations [34].

Additionally, due to SARS-CoV-2′s unprecedented position in the center of public discourse, both external events (e.g., the outbreaks in meat-processing plants) as well as highly visible pandemic preparedness measures such as additional surplus/emergency capacities for hospitals might have had a direct influence on the wider population’s adherence to the protective measures, including stay-at-home policies and social distancing. When summarizing these results, we can carefully formulate what emerged as ‘key tenets’ of our PD candidates’ pandemic response on a macro level: (i) the interviewed districts showed the willingness to act quickly, proactively and decisively in the novel pandemic situation. (ii) They were, furthermore, willing to deviate from standard procedure and willing to take risks by somewhat deviating from certain guidance they had received. (iii) PD candidates strived to keep as much information flowing towards them (situational analysis and reporting) and back from them to the wider public (coherent public communication), discovering unexpected positive influences on community compliance along the way. (iv) Furthermore, the utilization of social network effects seems to have been a helpful strategy.

While our study paradigm cannot conclusively prove that the identified strategies were causally linked to the successful SARS-CoV-2 mitigation, our results offer certain evidence that following these strategies is compatible with reaching favorable results and may be part of an effective set of strategies. Combined with the prior literature showing that the effectiveness of some of these factors had been proven in the context of other public health emergencies, this calls for further research aimed at ‘causally’ evaluating the effectiveness of the key tenets that emerged from our small sample of interviews in a broader study setting.

This need for further research is further compounded by the fact that pandemics and outbreaks had an impact on triggering disunities in societies. Jedwab et al.’s review reports the systematic disunity caused by pandemics and outbreaks until the emergence of SARS-CoV-2 [35]. They report that inclusivity of all community members is an important step for the control of infectious disease transmissions. This may reduce different forms of structural inequalities in societies [35]. Additionally, communicating the process of controlling a pandemic was proven to be effective in the past [36,37]. A recent systematic review by Mendez-Brito et.al. compared the different non-pharmaceutical interventions against COVID-19, and found that early intervention (including early risk communication and health protection dissemination) was highly effective [38].

### 4.2. Methodological Strengths and Weaknesses

In this study, we conducted a novel, unique, and holistic approach to identify successful practices for controlling the transmission of the SARS-CoV-2 pandemic [39]. To identify Positive Deviants, we performed a comparison between all districts regarding their ability to keep the reproduction rate low while considering structural, mobility- and weather-related differences between them. For this aim, we deployed a sequence of statistical methods to compare the actual reproduction rates with a district-dependent prediction from a statistical model fitted on all districts. Given the complexity of the comparison, stemming from the number of districts and different confounding factors, it would hardly be possible to perform such an analysis without automated methods. After this quantitative analysis, we conducted a qualitative investigation of the Positive Deviants’ pandemic response in the form of Focus Group Discussions, aimed at identifying factors that might have contributed positively to their relatively successful outcomes in our analysis [40].

In order to make fair comparisons between predicted and actual reproduction rates, we leveraged different types of data in an ARX(2,2) model. This model is time-dynamic by including the previous reproduction rates and exogenous influences in order to account for the dynamic nature of the pandemic in contrast to only using time-independent factors. Moreover, we aimed at deriving comparability between districts by adjusting the summer periods to the respective timeframes of schools’ summer vacation in each federal state. We further compared the districts not by utilizing the common approach of clustering (see Albanna and Heeks, 2019 for examples) but by employing a space-continuous selection of peers based on their similarity regarding socio-economic status and ruralness.

We wish to emphasize that the presented workflow is easily scalable with regard to the number of districts and temporal data points. In this lies a big advantage of the quantitative approach we used, especially in the context of DPPD: the qualitative analysis of the PDs is performed manually and can come with significant effort and time consumption, often days and weeks. For the identification of PDs, the acquisition of raw data can be laborious. However, once the data is in a usable format, upscaling of the number of districts and data points merely requires additional computation time in the quantitative methods, which, if the models are not too time-consuming, should lie in the range of seconds, minutes or, maximally, hours. We therefore separated the qualitative from the quantitative part so that we could easily scale up the former and maintain the cost of the latter by choosing the same number of PDs to be analysed.

However, there are also several limitations our study faces, particularly regarding the available datasets, on which our identification of the PD districts depended. Most importantly, the number of reported SARS-CoV-2 cases were influenced by the volume and allocation of testing resources. While the number of tests differed across districts, our calculation of reproduction factors from the ratio of consecutive weeks’ case numbers should eliminate any cross-sectional differences in numbers and allocation of tests. However, rapid temporal developments of testing volume and allocation could still have affected our analysis. While on the federal level, test numbers developed rather steadily across each of our periods of analysis [41], this remains a concern for which future research might strive to find a more definite solution. Still, even assuming the data represented the evolution of new infections perfectly, it is difficult to distinguish which developments stem from active behavioural changes and which represent statistical randomness. This holds especially true for districts with low absolute case numbers.

Furthermore, we did not include the non-PD districts in our qualitative interviews, but instead asked the participants to reflect on what differentiates their district’s response from most other districts as part of our interview guide. Nevertheless, we had to rely on our informants’ self-reported perceptions of the different practices among districts.

Additionally, we need to stress the multi-faceted nature of the SARS-CoV-2 pandemic, where any singular performance measure can only ever represent one aspect. We considered the weekly R0 of new infections as it was an important measure for the policy decision making in many countries [42]. However, different performance measures might be more appropriate in different contexts [43]. Together with potential shortcomings of the data, our PD districts were selected as PDs with respect to *our performance measure* and *our given data.*

### 4.3. Application of Our Approach in the Global South

While this was a data-driven project in a high-income context, DPPD stems from the context of global development/international collaboration. Therefore, we briefly want to discuss the implications of our methodology for low- and middle-income countries. This methodology’s adaptability relies on the availability and quality of the necessary data. This includes the availability of reliable local SARS-CoV-2 case numbers, data about individual mobility during the pandemic, weather data and data on ruralness and socio-economic status (Table 5). Our study thus further underlined the importance of the availability of standardized data, which has been emphasized by several international agencies, especially since the beginning of the SARS-CoV-2 pandemic [44]. If data on some of these quantities are not available, please note that our approach is modular in the sense that each step can be replaced or modified individually. As illustrated in Figure A1, we choose (1) a performance measure—in our case, the weekly reproduction rate. (2) We use data from different sources, which we use to (3) fit a time-dynamic model of future reproduction rates. (4) We identify PDs by comparing the difference between actual and predicted reproduction rates among structurally similar districts. This modular approach means that changing the performance measure, taking different confounding factors into account, using a different model structure or selecting PDs in a different way can be carried out in a way that fully maintains the rest of the workflow. Therefore, both for the identification of PDs regarding the spread of SARS-CoV-2 in other countries and in different contexts, we hope that this workflow, with all the details explained throughout this article, can be harnessed in other applications by replacing individual parts such as the performance measure, the data or model structure.

While we cannot guarantee that all presented solutions for pandemic inhibition emerging from the focus-group discussions are feasible in other countries, we believe that the applicability of the quantitative approach, which mainly depends on a sufficient existence of relevant data, is high.

## 5. Conclusions

We have tested a novel method to identify effective strategies in the pandemic context. This method included quantitative and qualitative methods. This method has great potential to be used internationally, especially in low- and middle-income countries, thanks to its reliance on publicly available datasets. The identified themes of fast-paced response, preparedness, situational analyses, containment, social network effects, communication and community compliance are consistent with the existing literature and thus warrant further research to enable reliable conclusions about potential causality.

## Figures and Tables

**Table 1:** Summary of the data sources.

Data Source (DS)	Data Type	Variables Used
1. RKI ^1^	SARS-CoV-2 case reports	Weekly reproduction factor (log scale).
2. Teralytics	Mobility data	Weekly number of trips both starting and ending in the respective district, i.e., internal trips and roundtrips. Weekly number of incoming trips to the respective district. Weekly incoming infection load, i.e., incoming trips multiplied by new cases per capita of the origin districts. All mobility features were standardized relative to population size in 2019.
3. DWD ^2^	Weather data	Weekly average temperature. Weekly average humidity. Weekly average precipitation per day. Weekly average hours of sunshine per day.
4. Landatlas study, Thünen-Institute	Structural data	Ruralness Index. Socioeconomic Status Index.

^1^—RKI: Robert Koch Institute, the German federal government agency and research institute responsible for disease control and prevention; ^2^—DWD: German Weather Service.

**Table 2:** Descriptive statistics. All timeseries data relates to weekly values from 9 March through 20 September 2020.

Variable	Mean	SD	Min.	PR-25	PR-50	PR-75	Max.
Log of R-factor	−0.02	0.78	−4.33	−0.53	0	0.45	3.37
R-factor	1.35	1.48	0.01	0.59	1	1.57	29
Internal trips per inhabitant	9.98	2.98	3.62	8.01	9.57	11.34	33.11
Incoming trips per inhabitant	4.28	1.78	0.7	2.98	3.99	5.37	12.62
Incoming infection load (10^−5^ per inhabitant)	42	53.6	0.48	11.06	24.77	53.12	898.93
Average temperature (°C)	14.78	4.9	0.16	11.64	15.51	18.18	25.41
Average humidity (%)	66.42	9.11	20.11	60.43	67.23	73.13	90.57
Average daily precipitation (cm)	1.58	1.98	0	0.11	0.87	2.36	21.72
Average daily sunshine (minutes)	473.67	145.73	34.03	354.92	474.22	594.8	801.24
Standardized socio-economic status index	0	1	−2.66	−0.55	0.06	0.65	3.45
Standardized ruralness index	0	1	−4.18	−0.6	0.39	0.72	1.44

Min. = minimum, Max. = maximum, PR-x = x-th percentile rank, SD = standard deviation. The column PR-50 corresponds to the variable’s median.

**Table 3:** Structural properties of the PD candidate districts. Names of those districts whose administration participated in a focus group discussion are in **bold**. Rur.—ruralness, SES—socioeconomic status, Pop.—population, Pop.-Dens.—population density (inhabitants per km^2^).

District	PD Timeframe	Province	Ranking during First Lockdown	Ranking during Summer Vacation	Rur.	SES	Pop.	Pop.-Dens.
**City of Koblenz**	**First Lockdown**	**Rhineland-Palatinate**	**1**	**68**	**−1.16**	**0.69**	**114052**	**1084**
**Lörrach**	**First Lockdown**	**Baden-Wuerttemberg**	**2**	**254**	**−0.03**	**0.97**	**228736**	**284**
City of Würzburg	First Lockdown	Bavaria	3	392	−1.29	0.49	127934	1460
**Gütersloh**	**Summer Vacation**	**North Rine-Westphalia**	**310**	**1**	**0.03**	**0.84**	**364938**	**377**
Dingolfing-Landau	Summer Vacation	Bavaria	291	2	0.83	1.99	96683	110
**Vechta**	**Summer Vacation**	**Lower Saxony**	**49**	**3**	**0.8**	**0.47**	**142814**	**175**

**Table 4:** Result summary of FGDs.

Theme	Characteristics
1. Characteristics of local administrations’ fast-paced SARS-CoV-2 emergency response	1.1. Action bias
	1.2. Agility in public administration
	1.3. Fill regulatory gaps/Courage to deviate
	1.4. Cross-organizational collaboration
2. Preparedness	2.1. Risk consciousness and early initiation of measures
	2.2. Emergency infrastructure and procurement
3. Situational analysis and reporting	3.1. Data Flow
	3.2. Analyses and Projections
	3.3. IT System in health departments
4. Containment	4.1. Strive to remain in control
	4.2. Shut down and isolate early and comprehensively
	4.3. Large scale testing
5. Communication	5.1. Reliable and trustworthy public communication
	5.2. Consistent health messages across all channels
6. Utilization of social capital	6.1. Personal networks (of decision makers)
	6.2. Role of social cohesion
7. Community compliance	7.1. Early preparedness measures reinforce risk consciousness among the population
	7.2. Proximity of major outbreak increases compliance

**Table 5:** Suggestions for data sources for the needed variables to test our methodology internationally.

Variable	Suggested Data Source	URL
SARS-CoV-2 case numbers	WHO Coronavirus Dashboard	https://covid19.who.int/table (accessed on 12 July 2021)
SARS-CoV-2 R0	WHO Coronavirus Dashboard	https://covid19.who.int/table (accessed on 12 July 2021)
Individual data mobility	Google Mobility	https://www.google.com/covid19/mobility/ (accessed on 12 July 2021)
Ruralness per country	World Bank—Rural access index	https://datacatalog.worldbank.org/dataset/rural-access-index-rai (accessed on 12 July 2021)
Socio-economic status *	World Bank ^	https://data.worldbank.org/country (accessed on 12 July 2021)

* For our German context, this was calculated from unemployment rate, mean salaries, mean income level, communal tax revenue, net population migration, residence vacancies, life expectancies, life expectancy at birth, and percentage of high school dropouts. ^ Not all data might be available, so, for low- and middle-income countries, we recommend using whatever comparable variables are available, provided that they can be considered a reasonable proxy for socio-economic status.

## Data Availability

Data and Python code are available online at https://github.com/gizdatalab/DPPD_Covid, accessed on 10 September 2021.

## Appendix B

**Table A1:** Regression weights from the ARX(2,2). Standardized to range [−1, 1].

Predictor Variable	Standardized β-Weights
Log-R0 t-1	−0.293
Log-R0 t-2	−0.081
Internal travel per capita week t	−0.337
Internal travel per capita week t-1	0.35
Incoming travel per capita week t	−0.668
Incoming travel per capita week t-1	0.694
Incoming infection load per capita week t	0.247
Incoming infection load per capita week t-1	−0.267
Average temperature week t	0.08
Average temperature week t-1	0.067
Average humidity week t	0.018
Average humidity week t-1	0.016
Average precipitation week t	−0.012
Average precipitation week t-1	0.043
Average daily sunshine week t	0.036
Average daily sunshine week t-1	−0.106

In Table A1, the regression weights from the ARX(2,2) model are shown. It would be desirable if the regression weights permitted an interpretation as to how the predictor variables influence the dynamics of the pandemic. We can observe that the predictor variables pertaining to mobility and incoming infection load have the highest weights and should therefore be the dominating ones in regard to the influences on the reproduction rate while the predictor variables describing weather effects seem to be of minor influence. The weights corresponding to the (logarithm of the) reproduction rate in weeks t-1 and t-2 have negative signs. This could indicate that when the reproduction rate is high, the population of a district reacts to this with the aim of decreasing the overall infection risk. Conversely, a period of low infection spread might lead to easing of official and personal protective measures, which in turn would lead to an increase in forward transmission.

Among the weights of the mobility-related factors we can see that the respective weights of a factor for week t and week t-1 are approximately opposite of each other. With this, the model equation contains approximations of the derivative—the rate of change over time—of these variables. E.g., for incoming infection load per capita, we find that the highly similar absolute weights show opposing signs for the t and t-1 terms. As a consequence, if the incoming infection load per capita increases from week t-1 to week t, then the reproduction rate should increase as well. In total, not the absolute incoming infection load is decisive but rather the way it changes in comparison to the previous week.

However, for internal and incoming travel per capita, the effect is opposite: increasing travel leads to lower estimates of the reproduction rate. This immediately raises suspicions as to whether the model coefficients actually uncover any causal relationship.

While the explanation that a lower number of people traveling into and around a district causes the reproduction rate to increase seems counterintuitive, a possible alternative explanation is that in districts that have the—rightful—reputation that it is safe to spend time in them, people travel around more freely. The decrease of the spread factor and the increase in travel would then be caused by the increased safety in regard to infection risk but would not stand in direct causation to each other.

Furthermore, we must consider multicollinearity effects, meaning that correlated predictor variables are assigned opposite weights while this has approximately the same effect on the dynamics as if one of the variables is assigned a small coefficient and the other is assigned a zero as coefficient. This is because for correlated factors, the terms approximately cancel out if weights are opposite to each other. For the three predictor variables corresponding to travel, we found high correlations between the respective values for week t and t-1 for the three variables. This makes interpretability of the regression weights even more difficult.

In order to increase the interpretability of the regression weights, it would require a sensitivity analysis or uncertainty quantification [45,46]. The former denotes an investigation of which predictor variables are vital to the model, i.e., leaving out which variable would significantly decrease the quality of the model. Plus, it denotes an analysis about which other sets of regression weights give a similar or even equal model quality but yield a different interpretation. The latter refers to an analysis of how incomplete or unreliable data perturb the result of the model fitting. A typical approach to this is Bayesian modelling [47].

In total, a possible interpretation of the determined regression weights is that human behavior counts more than weather-related influences and that the spreading factor reacts to the rate in which travel-related behavior changes over time. Moreover, the dynamics inside a district react to the reproduction rate of the previous weeks in a balancing way, predicting a high reproduction rate if the previous rate was low, and vice versa. As explained, without further analysis, we cannot make statements about the reliability of this interpretation. The aim of this article, however, is not the search for strategies that are drivers or inhibitors of the pandemic on the basis of a statistical analysis but through a qualitative analysis of the districts that were identified as positive deviants from the statistical model. To this end, the regression model serves its purpose of controlling for confounding influences, even if the individual weights are not readily interpretable.
